# Supplementary material for: Are carbon emissions trading and green financial instruments synergistic? -Comprehensive quantitative research based on content analysis
Source: PLoS One. 2024 Mar 7;19(3):e0298601. doi: 10.1371/journal.pone.0298601 (PMC10919601; doi:10.1371/journal.pone.0298601)
Supplement: S1 Data — (ZIP) [file pone.0298601.s001.zip › Data/地级市Z_F工作报告.zip!/║■─╧/╙└╓▌╩╨/└┤╫╘╣1⁄2╓┌║┼í╛┬φ┐╦╩2╛▌═°í┐_2019.docx]

各位代表：

现在，我代表市人民政府向大会报告工作，请予审议，并请各位政协委员提出意见。

一、攻坚克难，砥砺奋进，2018年工作取得重要进展

2018年，在市委的坚强领导下，在市人大、市政协的监督支持下，我们坚持以习近平新时代中国特色社会主义思想为指导，全面贯彻党的十九大精神，坚决落实党中央、国务院和省委、省政府各项决策部署，加快推进开放兴市、产业强市，经济社会发展取得新的全面进步。预计全市地区生产总值增长8.2%，规模工业增加值增长7.2%，固定资产投资增长10.7%，社会消费品零售总额增长10.3%，财政总收入增长8.3%，城乡居民人均可支配收入分别增长8.5%、9.3%。

三大攻坚全面发力。脱贫攻坚大局初定。全年预计脱贫13.5万人，贫困村出列267个，江华、新田2个国扶县摘帽，全市贫困发生率下降至1.3%，区域性整体贫困基本解决。易地扶贫搬迁“十三五”计划全面完成。教育、健康、住房等扶贫政策全面落实，贫困学生无一人因贫失学辍学，贫困群众县域内住院报销比例达到91.08%。“四扶四建”扶贫模式得到推广，“互联网+社会扶贫”、“四跟四走”产业扶贫、“万企帮万村”行动成效明显，774个贫困村村村建立主导产业，700余家扶贫车间吸纳1.2万余名贫困劳动力在家门口就业。风险防范积极有力。认真摸清政府债务底数，严格按照要求清理规范PPP和政府购买服务项目，加快推动平台公司市场化转型，严密防范和妥善处置非法集资案件，确保了金融债务风险总体可控和重点项目建设资金需求。深入推进“双百会战”，重拳开展“扫黑除恶”、“集打斗争”，积极稳妥处置群体性事件，不断深化基层平安创建和城乡网格化服务管理，全面加强法治信访和安全生产，社会大局保持和谐稳定。扫黑除恶、集打斗争、社会治安综合治理、信访工作综治考核排名全省第一，安全生产获评全省优秀单位，禁毒工作跻身全国先进。江华、宁远、双牌获评第四批全国法治县（市、区）创建活动先进单位。污染防治成效明显。认真抓好中央、省环保督察、审计署和中央、省委巡视交办环境问题整改，扎实开展国家生态文明建设示范市创建活动，全力打好蓝天、碧水、净土保卫战，深入推行“双河长制”，湘江保护和治理第二个“三年行动计划”全面完成，一批环保历史遗留问题和老大难问题得到有效解决。中心城区空气质量优良率达到84.3%、黑臭水体基本消除，县级以上集中式饮用水水源地水质全部达到Ⅱ类以上标准，境内江河湖库水质按水环境区划要求达标率97%，森林覆盖率达到65.33%。

产业强市步履坚实。“产业项目建设年”活动成果丰硕。“5个10”工程实施重大产业项目57个、重大科技创新项目33个、重大产品创新项目31个，引进500强企业32家、重大科技创新人才45名，500强企业引进数量排全省第二，宁远光电、恒伟药业、韬讯无人机人才引进3个项目获评全省“5个100”工程优秀项目，巨米智能、新辉开电子、中明新材料等一批高技术高质量项目实现当年洽谈签约、当年建成投产。园区升级步伐加快。全市工业园区完成基础设施投入102亿元，新建标准厂房106万平方米，新入园投资5000万元以上工业项目128个、竣工投产65个，新入统规模工业企业144家，百亿园区增至9个。江华经开区、宁远工业园晋升省级高新技术开发区，祁阳经开区获评国家新型工业化农副产品深加工（绿色食品）产业示范基地，新田工业园、冷水滩高科园获评国家林业产业示范园区。永州经开区招大引强成果丰硕，保利科技集团、新南方集团、古巴生物医药集团、深圳怡亚通等一批科技巨头纷纷入驻，“一谷两中心四基地”建设坚实起步，军民融合产业示范基地由省政府授牌。零陵烟厂在烟草行业生产经营整体下滑的形势下实现逆势上扬，完成产值39.6亿元、增长7.1%。现代农业稳步发展。新增高标准农田5万亩，大宗农产品生产稳面提质。新创市级农业标准化示范基地（场）65个，新认证“三品一标”农产品138个。省级以上龙头企业发展到52家，农产品加工业销售收入达到660亿元。全省首个市级全品类农业公用品牌“永州之野”成功发布，并跻身全国农业公用品牌影响力“十强”。回龙圩柑橘获第16届中国国际农产品交易会“十佳优质农产品”称号。圆满完成第三次农业普查。商贸物流势头良好。永州大市场、万商红商贸新城、愿景国际广场、零陵春天广场等一批重大商贸物流项目集中建成营业，全市限额以上商贸流通企业发展到596家，电商交易总额突破400亿元，江华获批全国电子商务进农村综合示范县。全域旅游持续升温。零陵古城、蓝山云冰山景区正式运营，永州华侨城卡乐文化旅游项目开工建设，祁阳杨梅湖智慧文旅生态新城、江华神州瑶族文化博览园等项目有序推进。全市新增3A级景区11个、星级乡村旅游区(点)47个，东安、江永、祁阳、零陵、江华获批第三批湖南省精品旅游线路重点县区。成功举办承办全省公祭舜帝大典、第十二届阳明山“和”文化旅游节、湖南省夏季乡村旅游节等重大节庆活动。全年游客接待量增长12%，旅游综合收入增长13.5%。文化产业快速发展，增加值增幅达到28.6%，新增规上文化企业52家，均排全省第一。金融保险稳健运行，全市新增表内外贷款211亿元，银担合作取得新突破。创新驱动能力增强。出台科技服务产业发展“十条措施”，财政科技支出、企业研发投入分别增长26.73%、69.23%，申请发明专利1224件，新认定高新技术企业78家，实施省以上科技计划项目75项、国家科技重大专项1项，获国家、省创新创业大赛奖项总数排全省第二。强力推进人才强市战略，设立人才发展专项资金5000万元，引进高学历人才837人、创新创业人才团队32个，新增1家院士工作站、3个博士及博士后工作站，创建省级以上科研平台6个。国家焊剂产品质量监督检验中心通过验收。获评“中国质量魅力城市”。

改革开放纵深推进。重点改革强力突破。以推进供给侧结构性改革为主线，全力抓好中央、省里部署的重大改革事项落实。新一轮机构改革稳步推进。“放管服”改革不断深化，市本级行政权力精简力度排全省第二，企业登记升级为“50证合一”。“互联网+政务服务”改革在全省推介，一体化平台基本建成，申请类事项网上可办率达到98%，市政务中心政务服务标准化国家示范点建设通过验收，省政务云异地灾备中心项目落户永州。积极推行项目建设联动机制、并联审批和行政审批归口管理制度改革，强化“五集中”审批服务，实行部门“一把手”到政务中心窗口坐班制度，营商环境持续改善。全省优化发展环境现场推进会在江华召开。农村综合改革深入推进，土地承包经营权确权登记颁证全面完成，集体产权制度改革试点有序开展，“两权”抵押全覆盖积极推进，供销综合改革成效明显。国资监管、行政执法体制、生态文明体制等改革迈出新步伐。开放兴市势头看好。全面实施“五大开放行动”，着力构建全面开放新格局，成功获批湘南湘西承接产业转移示范区，获评全省发展开放型经济优秀市。全年实际利用外资12.1亿美元、增长12%，内联引资314.8亿元、增长17.5%；完成外贸进出口总额17.5亿美元、增长29%，新增25家进出口实绩企业、62家进出口“破零”企业。湖南农副产品集中验放场（叠加永州公路口岸）启动运营，江华出口监管仓、进口保税仓建成使用，全市获批国家级水果蔬菜外贸转型升级基地。

城乡发展加速融合。基础设施建设取得新进展。湘桂铁路永州地区扩能工程、永州火车站提质改造工程加速推动，呼南高铁永邵客专进入开工前期阶段，兴永郴赣铁路、永贺铁路列入国家《中长期铁路网规划》，永清广高铁纳入《粤港澳大湾区规划纲要》，经停永州站高铁达到20对；零陵机场改造工程启动实施，新增航线5条，旅客吞吐量达到25.03万人次，同比实现翻番；衡永高速进入招商招标程序，零道高速、永郴高速列入全省高速公路建设中长期规划；S356沱涔一级公路、G207零陵朝阳大道、零双一级公路全面建成，湘江源至九嶷山公路开工建设；完成干线公路建设98公里、各类农村公路建设改造5099公里、危桥改造68座；湘江永州至衡阳千吨级航道改造工程前期工作完成。涔天河水库枢纽工程通过验收、灌区工程和毛俊水库建设进展顺利，何仙观水库列入省水利PPP试点项目，1座大型水闸、26座病险水库除险加固、5个小农水项目县建设任务全部完成。国华永州火电项目有序推进，50万千瓦风力发电项目全部开工，119兆瓦集中式光伏扶贫发电项目并网发电，中心城区生活垃圾焚烧年发电总量超过1亿度，宁远生活垃圾焚烧发电项目开工建设。完成762个行政村电网改造升级。完成277个行政村宽带网络升级改造，全市行政村宽带通达率达到99%，4G覆盖率达到96.3%。城镇提质扩容展现新风貌。全市城镇建成区面积拓展到215平方公里，城镇化率达到50.25%。中心城区实施重点城建项目58个，完成投资93亿元。永州大道地下综合管廊、二广高速永州东连接线改扩建工程推进速度加快，城南大桥、潇湘大道等项目进入冲刺阶段，冷东一级、冷祁一级市政化道路改造、九嶷大道北延伸段等项目进展顺利。市文化艺术中心、市民服务中心完成主体工程建设。永州师范高等专科学校建设基本完成。将军岭、文昌阁等高品质安置小区即将建成。2个BRT首末站投入使用，在全省率先实现“绿色公交”全覆盖。征地拆迁“百日攻坚”行动成效显著，重大项目落地明显提速。县城扩容提质和特色小镇建设持续推进，祁阳、道县、蓝山获评国家卫生县城，江华跻身全国文明城市提名城市，金洞“楠木康养特色小镇”入选国家森林小镇建设试点。乡村振兴战略带来新气象。全市新扩建1000亩以上特色产业基地34个，新创市级以上农业园区54个。新解决17.71万贫困人口饮水安全问题。完成120个省、市美丽乡村和1000个环境治理重点村建设任务，完成1000个村污水治理，新增卫生厕所28.8万户，全市农村生活污水处理率达到68%、生活垃圾集中处理率达到95%、保洁制度覆盖率达到99%、卫生厕所普及率达到75.8%，农村人居环境大为改观。

人民群众实惠更多。财政民生支出达到78%，重点民生实事全面完成。教育优先得到凸显。把城区学位建设作为“一号民生工程”，全年完成投资34.2亿元，通过“五个一批”方式新增城乡学位18.38万个，全市3451个超大班额全部消除。名师、名课、名校、名校长“四名”工程深入实施，高考上线人数创历史最大增幅。永州职业教育集团挂牌成立。以湖南科技学院为代表的高等教育内涵建设成效显著。社会保障不断加强。新增城镇就业6.63万人、农村劳动力转移就业4.28万人、市场主体5.2万户，城镇登记失业率2.32%，为近年最低。五项社会保险参保基本实现全覆盖，社会保障卡持卡人数达到528万。企业退休人员、机关事业单位退休人员人平养老金和城乡居民基础养老金分别提标到每月2269.43元、3863.07元、103元。城乡低保标准和特困人员供养标准分别提高到每月430元、272元、375元。城乡居民医保各级财政补助达到每人490元。救治救助无责任主体农民工尘肺病患者948名。每千名老年人口养老床位数达到36.5张。困难残疾人生活补贴和重度残疾人护理补贴每人每月提高到55元。完成240名0至6岁脑瘫儿童抢救性康复。对1400名智力、精神和重度肢体残疾人开展了托养服务。完成各类棚户区改造27206套、农村危房改造13798户。新建主城区特别是老城区地下排水排污管网110公里、改造48.4公里。新增城镇日供水能力18万吨。健康永州建设成效明显。中心城区医疗卫生“四个中心”建设有序推进，新完成38所乡镇卫生院、7所社区卫生服务中心和996所村卫生室标准化建设。农村适龄妇女“两癌”筛查、孕产妇免费产前筛查和农村65岁以上老人免费体检全面完成。新创学校标准化食堂200家，群众食品安全满意度排全省第一。计划生育工作向提素质、优服务转型。文化体育及各项事业全面发展。国家公共文化服务体系示范区创建深入推进，文化惠民不断加强，群众性文化活动蓬勃开展，在全省艺术节获奖数量创历史新高，宁远、道县、江永获批湖南省历史文化名城，1镇3村入选第七批国家历史文化名镇名村，成功申报61个中国传统村落。全民健身运动广泛开展，“大美永州”大穿越系列活动产生良好反响，荣获世界冠军1个、全国冠军5个。国防动员、国防后备力量建设和人民防空事业稳步推进，军民融合深入发展。民族宗教、外事侨务、新闻出版、统计、档案、史志、贸促、对台、气象、地震、老龄、慈善、残疾人、红十字会等工作取得新成效，工会、共青团、妇女儿童、关工委、科学普及等事业健康发展。

政府自身建设不断加强。坚持把政治建设摆在首要位置，自觉在市委的统一领导下开展工作，坚持重大工作、重大问题及时向市委报告，坚决执行落实市委的决策部署。全面执行市人大及其常委会的决议决定，自觉接受市人大及其常委会的法律监督、工作监督，自觉接受市政协的民主监督和社会舆论监督。办理人大议案2件、建议349件，政协提案286件、大会发言14件，学位建设、乡村振兴、优化发展环境等一批议案、重点建议和提案、发言办理成效明显。认真贯彻中央依法治国基本方略，依法行政稳步推进。扎实开展政府系统“执行力提升年”活动，制定出台并严格落实提升政府执行力“十条规定”和“十项改革”，着力治理政令不畅、办事拖拉、审批繁琐、考核评比过多过滥等突出问题，政府系统敢于担责、强于执行的能力有效提升。坚决整治违反中央八项规定精神的行为，切实强化监察监督和审计监督，转作风和反腐败斗争持续推进。

各位代表！过去的一年，我们经历了国际政治经济形势的风云变幻，经历了化解债务和环保督察等严峻考验，经历了部分骨干龙头企业遭遇市场严重下滑等特殊困难。面对这些严峻挑战，全市人民团结一心、迎难而进，各级各部门沉着应对、奋发作为，取得了比预计更好的结果。这些成绩的取得，是上级和市委科学决策、坚强领导的结果，是市人大、市政协大力监督和鼎力支持的结果，是全市人民凝心聚力、共同奋斗的结果。在此，我代表市人民政府，向全市645万父老乡亲，向中央、省驻永单位、驻永部队指战员，向所有关心、支持永州建设发展的同志们、朋友们，表示衷心的感谢和崇高的敬意！

各位代表！回顾2018年工作，我们清醒地认识到经济社会发展中存在的突出问题：一是高质量发展任重道远。产业基础仍然薄弱，骨干龙头企业和优质民营企业偏少；财政规模不大，质量不高，新兴税源培育不足，特别是市本级财政增收面临极大困难；创新驱动发展能力不强，研发投入偏少，科技人才和创新平台不足。二是产业园建设短板较多。区域性交通枢纽地位还没有形成，在承接产业转移竞争中仍然处于不利地位；缺乏“国字号”园区，大部分园区主导产业和产业链尚未形成；开放平台不足，通关功能不强；营商环境有待进一步改善。三是城镇化进程相对滞后。城镇化程度不高，特别是中心城区一体化进程不快，集聚力、辐射力、承载力不强，城乡统筹协调发展严重不足。四是惠民生任务仍然艰巨。脱贫攻坚虽然取得决定性进展，但巩固脱贫成果、提升脱贫质量仍需付出长期、艰苦的努力；能上学、能看病、能就业的问题虽然基本解决，但上好学、看好医、就好业的矛盾仍然突出；社会保障水平不断提高，但离人民群众的愿望和期待还有较大差距。五是转作风要求还需加强。一些干部在全面从严治党形势下，既有规矩意识不强的老问题，又暴露出担当精神不足的新问题，怕作为、不作为、慢作为、乱作为现象同时存在，形式主义、官僚主义依然严重。

这里，特别要向各位代表报告的是，由于政策和形势发生变化，特别是受一些不确定因素的影响，加上我们在工作上的欠缺，致使部分经济指标未能完成年初目标，部分重点项目未能如期实施，部分重点工作未能达到预期效果。对此，我们深感有负代表重托！在今后的工作中，我们将坚持问题导向，科学应对困难，始终牢记使命、心系人民，全力以赴把各项工作做好。

二、坚定信心，奋发作为，夺取2019年工作全面胜利

2019年是新中国成立70周年，是全面建成小康社会的关键之年。新的一年，我们既面临着经济发展方式的深刻变化、社会主要矛盾的深刻变化、外部环境的深刻变化等诸多变革，也迎来了高质量发展、改革创新、开放崛起等广阔发展空间；既面临着经济下行压力有所加大、不确定性因素有所增加、长期积累的风险隐患有所暴露等现实挑战，也迎来了“一带一路”建设深入推进、长江经济带发展、湘南湘西承接产业转移示范区建设、粤港澳大湾区建设等重大发展机遇；既面临着基础性、结构性、根本性问题等突出短板，也迎来了集聚力、辐射力、承载力日益增强等良好发展态势。今天的永州，正处在转型发展的攻坚期、跨越赶超的关键期、干事创业的黄金期。我们一定要深刻领会中央关于我国发展仍处于并将长期处于重要战略机遇期，呈现长期向好发展前景的重要判断，沉心静气看待当前形势，坚定有为抓好下步工作，共同为加快永州发展作出新的贡献。

根据市委部署，2019年全市经济社会发展的总体要求是：全面贯彻党的十九大和十九届二中、三中全会精神，以习近平新时代中国特色社会主义思想为指导，认真贯彻习近平总书记关于湖南工作的重要指示精神，统筹推进“五位一体”总体布局，协调推进“四个全面”战略布局，加强党对经济工作的全面领导，坚持稳中求进的工作总基调，坚持新发展理念，按照高质量发展的要求，以推进供给侧结构性改革为主线，深化市场化改革，扩大高水平开放，落实“巩固、增强、提升、畅通”八字方针，统筹推进稳增长、促改革、调结构、惠民生、防风险，坚定不移稳就业、稳金融、稳外贸、稳外资、稳投资、稳预期，大力实施“创新引领、开放崛起”战略，扎实推进开放兴市、产业强市，全力打好“三大攻坚战”，积极拓展“六大战役”和“四个提升”，加快建设创新开放新高地、品质活力新永州，为全面建成小康社会收官打下决定性基础，以优异成绩迎接新中国70周年华诞。

主要预期目标是：地区生产总值增长7.5%以上，规模工业增加值增长7.5%左右，固定资产投资增长10%左右，社会消费品零售总额增长11%左右，财政总收入增长7%左右，进出口总额增长15%以上，城镇居民人均可支配收入增长8%以上，农村居民人均可支配收入增长9%以上。环境保护、资源节约、安全生产等约束性指标完成省定任务。同时，按照高质量发展要求，更加注重以下指标：全社会研发投入占地区生产总值比重1.5%以上；产业投资占固定资产投资总额比重50%以上，其中工业投资占固定资产投资总额比重35%以上；税收占财政收入比重70%以上；单位地区生产总值能耗下降3%以上。

实现上述目标，工作中我们要牢牢把握以下几条：一是始终突出高质量发展这个鲜明主题。顺应经济从高速增长阶段转向高质量发展阶段的大势，真正实现发展思维从速度型转向质量型。二是始终突出三大攻坚战这个战略重点。坚持抓稳定就是抓发展、抓扶贫就是抓发展、抓治污就是抓发展，狠下决心解决发展中的突出矛盾和问题。三是始终突出产业项目建设年这个有效抓手。抢抓产业转移机遇，大抓产业项目建设，切实强化对产业项目建设的工作推动、政策支持、要素保障、考核奖惩，真正将产业强市战略落到实处。四是始终突出为民谋福祉这个根本目的。既尽力而为，又量力而行，着力解决人民群众普遍关心的突出问题，把实事办好，把好事办实，让发展成果更多惠及民生、温暖人心。五是始终突出提升执行力这个坚强保障。旗帜鲜明讲政治，扑下身子抓落实，以务实高效的作风服务于民，以勤政有为的形象取信于民，不断增强各级政府执行力和公信力。

主要工作任务是：

（一）紧紧围绕打造经济升级版，坚定推进产业项目建设

着力推动“5个10”工程新突破。坚持“大项目—大产业—大发展”路子，再掀“5个10”工程建设热潮。突出抓好50个重大产业项目、30个重大科技创新项目、30个重大产品创新项目实施，新引进30名科技创新人才、20个500强企业项目。围绕“5个10”工程推进，健全工作机制，完善配套政策，加强协调调度，强化考核督促，确保工程建设落地落实。

着力推动工业发展新突破。大力实施园区发展“5个100”工程，全年新增园区基础设施投资100亿元以上，新建标准厂房100万平方米以上，新签约入园投资5000万元以上工业项目100个以上，新入统规模工业企业100家以上，培育税收过200万元企业100家以上。扎实推动园区环境、动能、产业、服务、管理“五个升级”，加快建设绿色、创新、实力、智慧、活力园区。下大力气解决国家级园区“空白”问题，矢志不渝将永州经开区创建成国家级经开区，以江华、宁远省级高新区为依托谋划建设国家级高新区，新增1—2个省级高新区。突出抓好永州经开区“一谷两中心四基地”建设，着力推进亲水河科技谷、潇湘科技创新中心、中（国）古（巴）国际生物技术中心、天然植物提取高技术产业基地、保利军民融合产业示范基地、长丰汽车配套产业基地、华为大数据产业基地等重大项目建设，抓好与以湖南科技学院为依托的国内外知名大学和科研单位的校地企、产学研合作。把制造业发展放到突出位置，大力实施“聚链、补链、延链、强链”工程，加快发展以农产品加工、轻纺制鞋、矿产品加工等为主导的传统产业，快速壮大以汽车制造、电子信息、新材料新能源、植物提取物、大数据等为主导的新兴产业。加强产业联盟建设，市本级着力打造好生物医药、鞋业产业联盟，加快培育智能装备、电子信息等产业联盟。

着力推动现代农业新突破。围绕建设湘南优质农副产品供应基地，积极实施对接粤港澳大湾区“菜篮子”工程，突出抓好优质蔬菜基地扩面、食用菌生产基地扩能、生猪养殖基地提质以及35万亩红壤富硒有机茶产业化项目建设，新创农业标准化示范基地60个以上。大力发展农产品加工业，紧紧依托粮食、畜禽、柑桔、油茶、茶叶、蘑菇、生姜、香芋等优势产业，引进新上一批农产品精深加工企业，培育壮大一批龙头企业，全面提升农产品附加值和农业现代化水平。实施“永州之野”农业公用品牌提升工程，加快培育一批质量过硬的子品牌，加大向粤港澳大湾区的推广力度。切实加强农业平台建设，大力推动市农科园创建国家农业产业高新区；整合市农科所、柑科所、茶叶研究所、农机研究所等资源，组建永州现代农业产业研究院；加快永州国家食用菌研发中心建设，做好永州国家茶叶研发中心及检测中心筹建工作；着力引进一批集农产品批发、仓储物流、电商运营于一体的农产品综合服务平台。切实加强农产品质量安全，新增2个省级农产品质量安全县。

着力推动服务业新突破。深入推进文旅产业融合发展，围绕做大做强“古城名山”，全力支持零陵古城及配套项目建设，提升国家历史文化名城内涵，加快推进永州华侨城卡乐文化旅游项目建设，积极引进恒大文旅产业、好样传媒棠云影视小镇等项目，尽快形成中心城区文旅产业的集聚力和影响力；推动九嶷山舜帝陵景区创5A工作取得实质性突破，举办好全市公祭舜帝大典活动；抓好江华神州瑶族文化博览园、祁阳杨梅湖智慧文旅生态新城、蓝山云冰山景区二期、双牌国际慢城及阳明山大熊猫繁育教育基地等重点旅游项目建设。以市文化旅游开发投资公司为抓手，打造好对接粤港澳旅游市场的桥梁窗口，策划好湘粤桂边界区域精品旅游线路，建设好智慧旅游平台，实施好“旅游+”融合发展工程。实现全年游客接待量和旅游总收入增长10%以上。推进商贸流通“千百工程”和“优供促销”行动，培育打造“6个10”商贸流通载体工程，开工建设万达中心、王府井城市商业综合体等项目，着力引进1—2家大型智慧物流项目。抓好永州农产品电商孵化中心、潇湘创客汇电商产业园建设，力争新创1个全国电子商务进农村综合示范县。规范引导房地产业健康有序发展，坚决把房价稳定在合理水平。

（二）紧紧围绕激发创新创业新活力，坚定推进营商环境优化

加快推进关键领域改革。全面按期完成本轮机构改革任务，推动政府职能深刻转变，提升政务服务质量和效率。深入推进“放管服”改革，进一步精简审批事项，压缩审批时间。全面落实“证照分离”、“多证合一”、“证照联办”，推动“双随机、一公开”监管全覆盖。深化“互联网+政务服务”改革，加快实现一体化平台与省全面对接、市与县乡村全面联通，全力推进“马上办、网上办、就近办、一次办”和“帮代办、刷脸办、掌上办”，真正实现企业和群众办事“最多跑一次”。积极推进农村土地“三权分置”改革，稳步推进农村集体产权制度改革，完善农村土地流转体制机制，深化供销合作社综合改革。统筹抓好国企国资、要素市场化配置、自然资源管理、行政执法体制等基础性改革。围绕教育、就业、医疗、养老等群众关心关注的热点难点问题，精心推出一批有利于增强人民群众获得感的改革举措。

充分激发科技创新活力。抓好“加大全社会研发经费投入三年行动计划”，全年研发经费投入达到30亿元以上，市财政科技经费预算安排实现翻番，争取实施省以上科技计划项目40项以上，新认定高新技术企业50家以上，新申请发明专利1550件以上，增设院士工作站1—2家，创建省级以上研发“双创”平台2家，力争创建国家知识产权示范城市。实施“互联网+实体经济”行动，打造一批智能制造示范企业、示范车间。抓好“潇湘人才行动计划”落实，大力引进一批带项目、带成果的人才团队和高层次人才。切实做好引进人才的后续服务管理工作，实现引得进、留得住、干得好。

全力支持民营企业发展。切实打破准入门槛，国家未禁止的行业、领域全面向民营企业开放。切实抓好国家、省、市减负降本一揽子政策的落地落实，梳理编制优惠政策清单向社会公开，在税费、物流、用地、用能、用工等方面给予最大支持，坚决降低企业营商成本。切实加强财政、信贷、产业政策联动，建立健全民营企业贷款风险补偿机制，完善产业发展引导基金、金融机构新增贷款和企业成长奖励机制，做大做强市县区政府性融资担保公司，建立中心城区中小微企业“助保贷”平台，积极支持企业上市，实现县域村镇银行全覆盖，着力解决民营企业融资难融资贵问题。切实把民营企业和企业家的事当作政府自己的事，理直气壮为民营企业发展保驾护航。实施中小企业服务平台全覆盖工程，健全领导干部包联重点企业制度。以开展“优化营商环境执行年”活动为抓手，盯住重点部门、重点问题、重点企业，突出解决企业“难、烦、怕”的问题。依法严厉打击侵害民营企业权益的违法犯罪行为，切实保护好企业家人身和财产安全。加快培育一批优质民营企业和优秀民营企业家，撑起民营经济发展的“参天大树”。

（三）紧紧围绕打好全面小康决胜战，坚定推进精准脱贫攻坚

咬定目标任务。按照省定计划和要求，全年减少贫困人口5万人，贫困村出列32个，贫困发生率下降到1%以内，除兜底保障对象外，基本消除绝对贫困，贫困村全部脱贫出列。

抓实产业扶贫。把2019年作为扶贫产业建设年，加大资金支持力度，推动贫困地区扶贫产业发展壮大、提质增效。推进“万企帮万村”行动，结合乡村振兴战略因地制宜发展特色种养、乡村文化旅游、农村电商等产业，促进产业就业融合发展。支持有条件的乡镇创办扶贫产业园，引导更多企业在贫困村建立“扶贫车间”，力争实现“村村有主导产业、户户有就业项目”，构建贫困人口稳定增收、持续发展的良性机制。切实做好扶贫小额贷款风险防范工作。

攻克深度贫困。进一步加大对深度贫困村的帮扶力度，优先实施水利、交通、电力、信息等基础设施项目，优先实施教育、文化、卫生、科技等公共服务项目，优先实施扶贫产业项目，优先安排退耕还林、生态修复、森林管护等项目，同时在人才支持、队伍帮扶、社会帮扶等方面给予倾斜。年内所有深度贫困村实现通硬化路和完成危房改造任务。健全完善兜底保障政策，加大重病重残困难群众保障力度，对符合条件人员实现“应扶尽扶、应保尽保”。

巩固脱贫成果。坚持脱贫不脱政策、脱贫不脱帮扶、脱贫不脱监管，对摘帽县、出列村和已脱贫人口给予持续帮扶，精准落实教育、医疗、住房等到村到户到人的保障政策。统筹兼顾非贫困村、非贫困人口的产业帮扶、基础设施建设。建立防止返贫的研判预警机制、动态管理机制和跟踪帮扶机制，最大限度减少和防止因残、因病、因灾返贫。及早研究解决那些收入水平略高于建档立卡贫困户的群体缺乏政策支持等新问题，提高脱贫攻坚“夹心层”群体的生活水平。大力推进“四扶四建”扶贫模式，充分激发贫困群众内生动力。加强贫困村基层组织建设，筑牢巩固脱贫成果的坚强堡垒。

（四）紧紧围绕保护美丽湘江源，坚定推进环境污染防治

打好蓝天保卫战。深入推进国家生态文明建设示范市创建工作，积极创建“大气环境质量达标城市”。大力实施国土绿化行动，完成人工造林27万亩，封山育林28万亩，确保全市森林覆盖率稳定在65%以上。持续开展“散乱污”企业整治，抓好10个挥发性有机化合物治理项目，严格实施燃煤锅炉清洁能源改造，大力推广新能源汽车，狠抓建筑、道路扬尘污染治理，完善空气质量监测体系。年内中心城区空气质量优良率达到85%以上，县城达到87%以上。

打好碧水保卫战。大力实施湘江保护和治理第三个“三年行动计划”，突出抓好湘江源头流域污染综合治理、农村养殖污染治理、资源开发利用造成的水环境污染治理以及既有污水处理厂提标扩能、滨江新城污水处理厂及8个乡镇污水处理厂新建、9处县级以上城市黑臭水体治理等项目实施，压实各级河长责任，持续开展河库管理保护“清四乱”专项行动，全面开展饮用水水源地集中整治，探索流域水污染治理新方式，逐步在县区间开展生态补偿机制试点，对水环境质量实行分段把关、分段考核、分段补偿，确保全市市级水功能区水质达标率100%，县城以上饮用水水源地水质达到Ⅱ类比例100%。

打好净土保卫战。积极实施土壤环境保护工程，扎实保护好“山水林田湖草”，抓好湘江两岸“小微湿地”试点，高标准、高品质建设1—2个国家湿地公园，确保全市湿地保护率稳定在72%以上。加强矿山生态修复与治理，突出抓好零陵锰矿区生态修复、东安重金属污染治理等项目实施。加快生活垃圾焚烧处理设施建设，年内基本建成宁远、开工建设道县垃圾焚烧发电项目，推进永州静脉产业园做大做强。强化固体废物污染防治，抓好农业面源污染治理，加快推进垃圾分类处理和资源化利用，坚决管好永州每一寸土地。

（五）紧紧围绕筑牢发展防火墙，坚定推进重大风险防范

严防金融债务风险。严格落实政府隐性债务风险化解实施方案，强化债务限额管理和预算管理，大力推进平台公司市场化转型。加强与金融机构的沟通协调，积极采取借新还旧、展期、续贷、降息等方式，缓解化债压力。严厉打击恶意逃避金融债务和非法集资行为，确保“三保”资金不断裂、重大金融债务风险事件不发生。科学处理好抓化债与促发展的关系，决不能因化解债务造成资金链断裂，造成半拉子工程，造成束手不干事。深入实施“新财源培植工程”，加快建立重点财源企业库，开展重点财源企业精准帮扶行动。加快推进中心城区财政体制调整，充分调动市区两级抓财、培财、理财的积极性。

严防安全生产风险。严格落实“党政同责、一岗双责、失职追责”要求，全面强化党政领导责任、属地管理责任、部门监管责任、企业主体责任。强力推进非煤矿山、危险化学品、道路和水上交通等重点行业领域专项整治，落实重大事故隐患治理“一单四制”，坚决防范和遏制重特大事故发生。切实抓好建设工程质量安全管理，落实监管重心前移。加强应急管理，科学应对处置各类突发事故，提升综合防灾减灾救灾能力。

严防社会稳定风险。以新中国成立70周年安保维稳工作为主线，严密防范、严厉打击境内外敌对势力的渗透破坏活动，坚决捍卫以政权安全、制度安全为核心的国家政治安全。始终坚持严打方针不动摇，持续深入开展“集打斗争”和“扫黑除恶”专项斗争，着力将打击锋芒对准八类严重刑事犯罪，对准黑拐枪、盗抢骗、黄赌毒、食药环、电信网络诈骗、“三强三堵”等危害性大、牵涉面广的违法犯罪。扎实开展新一轮禁毒人民战争，全面巩固全国禁毒示范城市创建成果。加强治安防控体系建设，深入推进“平安创建”，全面开展智慧安防村庄、小区、单位创建活动。深化大数据警务战略，构建智慧警务新体系。深入推进信访法治化建设，大力开展“积案化解攻坚”行动和“三无”创建活动，建立群众诉求协办员机制，依法依规及时就地解决群众合理诉求。推动公共法律服务城乡全覆盖，加快基层司法所规范化建设，不断提升普法工作实效。

（六）紧紧围绕加速区域一体化，坚定推进城乡融合发展

完善城乡基础设施。抓紧抓实呼南高铁永邵段、永清广高铁、兴永郴赣铁路、洛湛铁路永贺段改造项目前期工作，加快推进湘桂铁路永州地区扩能工程建设，全面完成永州火车站提质改造，争取新增3—4趟高铁停靠。分段推进衡道高速项目建设，力争开工建设衡阳至零陵段，抓好零陵至道县段前期和招商工作，积极协助省里推进冷水滩至新宁高速公路前期、道县至连州高速公路规划工作。开工建设湘江永州至衡阳千吨级航道改造永州段一期工程，积极协助部、省推进湘桂运河项目前期规划工作。完成零陵机场跑道改造项目，争取新开通2—3条航线。加快推进潇湘城市群之间市政化快速连接道路建设，启动宁远至道县、道县至江华一级公路建设，抓紧九嶷山、湘江源、阳明山、舜皇山等景区配套道路项目实施。开展城乡客运一体化试点，启动“公交都市”创建工作。力争分别创建1个国家和省级“四好农村路”示范县区。扎实推进涔天河水库灌区工程、毛俊水库建设，积极做好零陵何仙观水库、宁远龙形头水库和中心城区应急备用水源项目前期工作，完成21座病险水库除险加固，推进高效节水灌溉、水土保持、中小河流治理、小水电整治、城市防洪等民生水利事业，加强对中小型水库渠道的建设和维护。加快国华永州电厂建设步伐，推进城乡电网建设改造，实施工业园区供电强基工程。加快“气化永州”长输管线建设，大力发展清洁新能源。加快“智慧永州”建设，推进“电信普遍服务试点”、“信息通信基础设施能力提升三年行动计划”等项目有序实施。

提升城镇功能品质。坚持规划引领，加快编制全市国土空间规划，编制完善各类专项规划，细化规划管理，保障城乡规划有序实施。围绕做大做强中心城区，加速滨江新城崛起，重点推进华侨城卡乐文旅项目和文旅综合体项目片区的配套道路建设，抓紧完成永州大道地下综合管廊及后续配套工程等在建项目建设，加快启动市文化艺术中心、市民服务中心两大片区开发。以实施“城市双修”为抓手，推进中心城区综合提质，大力推动湘江西路永州大桥至潇湘大桥段、湘江东路宋家洲大桥至潇湘大桥段建设，加快推进逸云路城中村改造、宋家洲安置小区及市园林局片区、长丰片区、湘江西路片区等棚改项目建设，启动高铁新城前期工作，加快铜锣湾体育文旅公园、市体育中心提质改造等项目建设，开工建设一批商业综合体和高品质住宅小区。加快智能停车场、充电基础设施等新型市政设施建设。深化城管体制改革，全面推行路（街）长制，完善数字城管平台。深入推进控违拆违和征地拆迁攻坚行动，加大闲置土地处置利用力度，做好做活土地资源文章。把县城建设作为推进新型城镇化和县域经济发展的战略重点，加强市政公用设施建设，实施环境容貌综合整治，深度推进产城融合。积极开展创新型城市建设，争创一批国家和省级城市品牌。推动建制镇彰显特色、竞相发展，每个县区重点抓好2—3个旅游、文化、边贸、加工型特色小镇，金洞、回龙圩分别抓好森林康养小镇、柑橘小镇建设，让更多特色城镇穿点成线、串连成珠。

加快乡村振兴步伐。全面落实乡村振兴战略规划，深入学习浙江“千万工程”经验，加快推进“十百千万”工程，着力打造一批具有影响力、引领性的乡村振兴示范区。突出以产业振兴带动乡村全面振兴，大力推进农工融合、农旅融合、农商融合，规划申报1—2个国家或省级田园综合体，打造一批特色农业小镇和特色文化村。大力改善农村人居环境，新创省、市美丽乡村示范村60个以上，完成市级农村环境整治重点村450个以上，抓好300个村的污水治理，推进“厕所革命”。大力实施乡村人才振兴计划，积极引导企业主体和工商资本进农村，加快培育新型职业农民，支持鼓励大学生、进城务工人员、退役军人等各类人才投身乡村建设，探索推进“新乡贤”回村助力乡村振兴。加强乡风文明建设，广泛开展改陋习树新风活动，大力培育文明乡风、良好家风、淳朴民风。

（七）紧紧围绕建设承接产业转移示范区，坚定推进扩大对外开放

狠抓精准招商。抢抓湘南湘西承接产业转移示范区建设机遇，深入实施“五大开放行动”，积极争当承接产业转移排头兵。加强与粤港澳大湾区、长三角等发达地区的园区、商会、企业对接合作，紧盯产业转移意向园区和企业，做好“母亲式”服务。加强对三类500强和上市企业产业布局、投资趋向、发展需求的跟踪研究，精准制定政策，主动上门对接，全力招大引强。围绕主导产业做大做强、传统产业转型升级和新兴产业发展壮大，大力实施产业链招商。充分发挥异地永州商会和永州异地商会的作用，做活以商招商文章，引导支持永州籍在外经商办企业成功人士回乡组建投资人协会。扎实抓好重点节会招商，积极开展全方位、多层次的项目对接洽谈活动，打造1—2个具有鲜明永州特色的高水平节会品牌。实现全年实际利用外资13亿美元以上，内联引资350亿元以上。

完善开放平台。把平台建设作为推动开放型经济发展的重大基础性工程来抓，统筹建设面向粤港澳大湾区产业转移的公共服务平台、科技创新平台、投融资平台等，努力实现无缝对接，全力提高营商效率和贸易便利化水平。设立永州海关，扎实抓好湖南农副产品集中验放场、县区出口监管仓和进口保税仓、国家级出口食品农产品质量安全示范市、外贸综合服务平台、中小商贸企业服务平台、国家级果蔬外贸转型升级基地、跨境电商平台、电商公共服务平台、湘南鞋类交易平台、物流中心平台等10个开放型经济平台建设。通过3年左右的努力，构建健全完善、高效便捷的开放型经济平台体系。

拓展外贸市场。积极应对国际贸易形势变化，大力开拓新兴贸易市场，持续推动外贸“破零倍增”行动，力争新增“破零倍增”企业50家以上。大力推动“永品出境”、“永企出海”，有针对性地组织企业参加各种经贸活动和境外展会，支持优势产业、优秀企业、优质产品走出去。培育壮大一批生产型外贸企业，加快发展跨境电子商务、外贸综合服务等贸易新业态新模式，保持外贸稳定增长。实现外贸进出口总额20亿美元以上。

（八）紧紧围绕增进人民群众幸福感，坚定推进民生福祉改善

着力抓好十大民生工程建设。（1）把农村安全饮水作为今、明两年为民办实事工作的重中之重，两年投资38亿元，实现全市农村安全饮水全覆盖和自来水“村村通”。今年完成投资20亿元，实现农村自来水普及率、水质达标率80%以上，供水保证率90%以上。（2）持续推进城区学位建设“民生一号工程”，全年完成投资31.4亿元，新建学位6.41万个，消除3286个大班额，实现城区学位建设三年攻坚行动圆满收官。（3）加快城乡幼儿园建设，全市新增公办幼儿园和普惠性民办幼儿园22所，缓解城乡幼儿入园难、入园贵问题。（4）加强农村公路建设改造，完成窄路加宽557公里、自然村通水泥(沥青)路建设1740公里。（5）推进农村电网改造升级工程，全年投资7.8亿元，改造511个行政村。（6）推进保障性安居工程建设，全市实施棚户区改造4500户、面积45万平方米，完成年度投资13.5亿元。（7）完成72个乡镇卫生院、15个社区卫生服务中心和1069个村卫生室标准化建设，实现基层标准化医疗卫生机构全覆盖。（8）抓好创建国家公共文化服务体系示范区文化设施项目建设，着力打造市文化艺术中心、规划馆、图书馆、博物馆、科技馆等市级标志性工程，加快县区公共文化服务设施建设。（9）深入实施主城区特别是老城区地下排水排污系统建设攻坚工程。（10）加快城区公共停车场、停车泊位、人行天桥、地下人行通道建设，加强小区整治，改善市民出行条件和居住环境。

不断提高社会保障水平。高度关注就业形势，全面落实稳就业政策，全年新增城镇就业5.7万人、农村劳动力转移就业4万人。大力开发特色就业岗位，做好残疾人、低保对象、零就业家庭等困难群体就业援助工作。切实维护劳动者合法权益，保障民工工资足额按时发放。健全各项社会保障制度，稳步提高最低生活保障和特困供养标准。完善城乡大病保险制度，提高城乡居民大病保险筹资标准、支付比例和年度补偿限额。抓好临时救助工作，支持慈善事业发展，关爱失独家庭、农村留守人员，重视老龄事业发展，加强残疾人康复服务。

全面发展各项事业。落实教育优先发展战略，依法保障教师待遇地位，深入推进“四名”工程，稳步增加对学前教育、农村贫困地区儿童早期发展、职业教育、特殊教育的投入，推动教育公平、优质、均衡发展。加大校外培训机构整治力度，切实减轻学生学业负担。全面完成江华、新田“芙蓉学校”省定建设任务，确保永州师范高等专科学校如期建成使用。推进健康永州建设，巩固深化医药卫生体制改革，扎实抓好医疗、医保、医药“三医联动”改革，全面开展好医德、好医术、好医生“三医联评”活动。完善分级诊疗制度，大力培养全科医生和乡村医生。推进医联体、医共体和县域医疗集团建设，积极推行以按病种付费为主的医保支付方式改革，把更多救命救急的好药纳入医保。加快推进中心城区医疗卫生“四个中心”建设，实现所有县区二甲公立医院全覆盖。强化急性传染病联防联控。传承发展中医药事业。鼓励支持社会力量发展健康产业。大力开展食品药品领域突出问题专项整治，新创学校（单位）标准化食堂200家。打好打响国家历史文化名城品牌，加大历史文化名城名镇名村保护力度，深入实施文化惠民工程和文化名家培育工程，打造2—3个国家级文化活动品牌。推进媒体融合，加快建立市县区融媒体中心。启动《永州市志》总纂，完成部门志编修。广泛开展全民健身运动，完善全民健身公共服务体系，加大青少年体育资金投入。大力弘扬社会主义核心价值观，深化各类精神文明创建活动，加快建设新时代文明实践中心，重视家庭家教家风建设，努力创建全国文明城市。扎实抓好第四次全国经济普查、第三次国土调查工作。认真做好民族宗教、外事侨务、统计、档案、保密、气象、地震、人防、水文等工作。充分发挥工会、共青团、妇联、关工委等群团组织作用。大力支持国防和军队改革，推进军民融合深度发展，加强国防后备力量建设，做好军转干部和退役士兵接收安置工作，营造全社会尊崇军人的浓厚氛围。

三、强化执行，狠抓落实，持续推动人民满意政府建设

坚持以政府系统执行力建设为引领，深入落实提升执行力“双十条”，在打造讲政治、敢担当、有作为、守清廉的人民满意政府上迈出新的更大步伐。

坚持用政治过硬的标准统揽执行落实。毫不动摇地把学习贯彻习近平新时代中国特色社会主义思想作为长期政治任务，坚定“四个自信”，增强“四个意识”，践行“两个维护”，始终保持政治上的清醒和坚定。毫不动摇地将政府工作置于市委领导之下，自觉维护市委权威，坚决落实市委决策。毫不动摇地加强政府系统党的建设，严格执行新形势下党内政治生活若干准则，坚决同不良现象和行为作斗争，营造风清气正的良好政治生态。

坚持用民主法治的理念引领执行落实。主动接受市人大及其常委会的法律监督和工作监督，坚决执行市人大及其常委会的决议决定，认真办好人大代表建议、意见，积极回应人大执法检查和专题询问。自觉接受市政协和各民主党派、工商联、无党派人士的民主监督，认真办好政协提案和大会发言，虚心听取采纳各方面的意见建议。实行市政府主要领导定期调度建议提案办理工作制度，切实提高办理质量。积极推进信息公开，打造阳光政府。大力推行严格、规范、公正、文明执法，加快法治政府建设步伐。

坚持用担当有为的精神狠抓执行落实。集中精力抓重点工作，倾注更多心血，汇集更多政策，整合更多资源，推动重大决策、重大任务、重大项目落实落地。创新思路抓亮点工作，立足永州实情，对标先进地区，善于学习借鉴，敢于先行先试，努力打造一批体现永州特色、展现永州形象的工作亮点。精细管理抓日常工作，转变粗放式管理的惯性思维，加强精细化管理、扁平化管理，做到各项工作执行有序、推进有力、落地有声。全面落实市委进一步激励广大干部新时代新担当新作为的部署要求，旗帜鲜明为敢于担当者鼓劲撑腰，努力锻造一支忠诚干净担当的高素质干部队伍。

坚持用务实清廉的作风保障执行落实。始终保持政治定力，严格落实“一岗双责”，加大对脱贫攻坚、公共资金、国资国企、项目招投标、公共资源交易等重点领域的监管，严肃整治行业不正之风。坚决落实中央八项规定和实施细则，严守政治纪律和政治规矩，紧盯不敬畏、不在乎、喊口号、装样子的问题，驰而不息纠正和整治四风，坚决破除形式主义、官僚主义。大力削减文山会海，压缩检查评比，切实为基层干部减负松绑。不断强化监察监督、审计监督，实现对公共资金、重大投资、重点工程监督全覆盖。严格压缩一般性支出，挤出资金保民生、保重点，努力用各级政府的“辛苦指数”换取人民群众的“幸福指数”。

各位代表，踏上新征程，奋进新时代。让我们更加紧密地团结在以习近平同志为核心的党中央周围，在市委的坚强领导下，以更加饱满的激情、更加有力的担当、更加勤勉的作为，励精图治，开拓进取，为实现全面建成小康社会的宏伟目标而努力奋斗！
